# Diagnosis rates, therapeutic characteristics, lifestyle, and cancer screening habits of patients with diabetes mellitus in a highly deprived region in Hungary: a cross-sectional analysis

**DOI:** 10.3389/fendo.2024.1299148

**Published:** 2024-05-01

**Authors:** Kata Pártos, David Major, Norbert Dósa, Vince Fazekas-Pongor, Adam G. Tabak, Zoltán Ungvári, Ildikó Horváth, Ildikó Barta, Éva Pozsgai, Tamás Bodnár, Gergely Fehér, Zsófia Lenkey, Mónika Fekete, Zsolt Springó

**Affiliations:** ^1^ Department of Public Health, Faculty of Medicine, Semmelweis University, Budapest, Hungary; ^2^ Department of Internal Medicine and Oncology, Faculty of Medicine, Semmelweis University, Budapest, Hungary; ^3^ Department of Epidemiology and Public Health, University College London, London, United Kingdom; ^4^ Vascular Cognitive Impairment and Neurodegeneration Program, Oklahoma Center for Geroscience and Healthy Brain Aging, Department of Biochemistry and Molecular Biology, University of Oklahoma Health Sciences Center, Oklahoma City, OK, United States; ^5^ Department of Health Promotion Sciences, College of Public Health, University of Oklahoma Health Sciences Center, Oklahoma City, OK, United States; ^6^ International Training Program in Geroscience, Doctoral School of Basic and Translational Medicine/Department of Public Health, Semmelweis University, Budapest, Hungary; ^7^ The Peggy and Charles Stephenson Cancer Center, University of Oklahoma Health Sciences Center, Oklahoma City, OK, United States; ^8^ Ormansag Health Center, Ormánság Egészség Központ (OEKP), “AZ ORMANSÁG EGÉSZSÉGÉÉRT” Nonprofit Kft., Sellye, Hungary; ^9^ Department of Public Health Medicine, University of Pécs Medical School, Pécs, Hungary; ^10^ Department of Primary Health Care, University of Pécs Medical School, Pécs, Hungary; ^11^ Department of Anesthesia, Luzerner Kantonsspital, Sursee, Switzerland; ^12^ Centre for Occupational Medicine, Medical School, University of Pécs, Pecs, Hungary; ^13^ Department of Primary Health Care, Medical School, University of Pécs, Pecs, Hungary; ^14^ Heart Institute, Medical School, University of Pécs, Pécs, Hungary; ^15^ Clinical Medicine Doctoral School, Department of Public Health Medicine, University of Pécs Medical School, Pécs, Hungary

**Keywords:** diabetes mellitus, diabetes diagnosis, undiagnosed diabetes, diagnosed disease, social deprivation

## Abstract

**Introduction:**

Low socioeconomic status affects not only diagnosis rates and therapy of patients with diabetes mellitus but also their health behavior. Our primary goal was to examine diagnosis rates and therapy of individuals with diabetes living in Ormánság, one of the most deprived areas in Hungary and Europe. Our secondary goal was to examine the differences in lifestyle factors and cancer screening participation of patients with diagnosed and undiagnosed diabetes compared to healthy participants.

**Methods:**

Our study is a cross-sectional analysis using data from the “Ormánság Health Program”. The “Ormánság Health Program” was launched to improve the health of individuals in a deprived region of Hungary. Participants in the program were coded as diagnosed diabetes based on diagnosis by a physician as a part of the program, self-reported diabetes status, and self-reported prescription of antidiabetic medication. Undiagnosed diabetes was defined as elevated blood glucose levels without self-reported diabetes and antidiabetic prescription. Diagnosis and therapeutic characteristics were presented descriptively. To examine lifestyle factors and screening participation, patients with diagnosed and undiagnosed diabetes were compared to healthy participants using linear regression or multinomial logistic regression models adjusted for sex and age.

**Results:**

Our study population consisted of 246 individuals, and 17.9% had either diagnosed (n=33) or undiagnosed (n=11) diabetes. Metformin was prescribed in 75.8% (n=25) of diagnosed cases and sodium-glucose cotransporter-2 inhibitors (SGLT-2) in 12.1% (n=4) of diagnosed patients. After adjustment, participants with diagnosed diabetes had more comorbidities (adjusted [aOR]: 3.50, 95% confidence interval [95% CI]: 1.34–9.18, p<0.05), consumed vegetables more often (aOR: 2.49, 95% CI: 1.07–5.78, p<0.05), but desserts less often (aOR: 0.33, 95% CI: 0.15–0.75, p<0.01) than healthy individuals. Patients with undiagnosed diabetes were not different in this regard from healthy participants. No significant differences were observed for cancer screening participation between groups.

**Conclusions:**

To increase recognition of diabetes, targeted screening tests should be implemented in deprived regions, even among individuals without any comorbidities. Our study also indicates that diagnosis of diabetes is not only important for the timely initiation of therapy, but it can also motivate individuals in deprived areas to lead a healthier lifestyle.

## 1 Introduction

Diabetes mellitus is one of the four most important non-communicable diseases affecting approximately 537 million adults worldwide, and their number is projected to further increase in the upcoming decades (1, 2). Although the relationship between social disadvantage and ill health is complex, evidence suggests that non-communicable diseases affect groups from lower socio-economic status more excessively than others (3). This socioeconomic gradient is also apparent for diabetes (4). The impact of poverty on health is multifaceted and mediated by the lack of material resources, higher psychosocial stress, more frequent occurrence of risky health behaviors, unhealthy living conditions, and limited access to high-quality health care (5). Socioeconomic status can be assessed either based on personal characteristics, such as income or education level, or regional measures, as indicated by deprivation indices (6). Both individually assessed low socioeconomic status and socioeconomic status conveyed by regional deprivation indices increase the risk of developing diabetes and related complications (4, 7, 8).

Ormánság is an area located in the Southern Transdanubian region of Hungary and is often considered as one of the most deprived regions within the European Union (EU) (9). The inhabitants of Southern Transdanubia make up 9% of the Hungarian population, however their contribution to the national gross domestic product is only 6.1% with a purchasing power standard of only €15,200 compared to the EU average of €29,900 (10). The Ormánság consists of 42 communities with a population of approximately 15,000 inhabitants (11). The median age in this area is 45.5 years, which is one of the highest in Hungary (11). The employment rate varies by settlement from 13.2% to 40.7%, whereas unemployment rates range from 1.2% to 26.3%. (11). The proportion of minorities, mostly Romani, also differs between settlements from 0.82% to 72.4% (11).

The “Ormánság Health Program” was launched in 2019 to improve the health and lifestyle of individuals in this highly deprived region of Hungary. Since low socioeconomic status may affect diabetes diagnosis rates, therapy, and health-related behaviors, our goal was (1) to examine the proportion of patients with diagnosed and undiagnosed diabetes and their therapeutic characteristics, and (2) to examine how individuals affected with diagnosed and undiagnosed diabetes differed from healthy individuals regarding their lifestyle and health-related behaviors, such as participation in cancer screening tests, utilizing data obtained from the “Ormánság Health Program.”

## 2 Materials and methods

### 2.1 Participants

Our study is a cross-sectional analysis using data from the “Ormánság Health Program” (12). The “Ormánság Health Program” was launched in December 2019 financed by the Hungarian Ministry of Human Resources and was completed in February 2023. The goal of the program was to promote the prevention of non-communicable diseases in the Ormánság by improving lifestyle, therapy adherence, screening participation, and health literacy of inhabitants. To achieve this, individual and group counseling sessions were organized for participants along with communitywide programs with on-site consultation and screening tests for cervical cancer and breast cancer. Participants were mostly recruited online. Participation was voluntary. To decrease healthy volunteer bias, local general practitioners, nurses, and community members also reached out directly to individuals who are traditionally harder to reach. Participants filled out a self-reported questionnaire and then took part in lifestyle medicine consultations. When completing the questionnaire, blood pressure was measured and capillary blood samples were also collected to measure certain parameters, such as blood sugar and cholesterol. The program was approved under 45312-5/2019/EGST.

### 2.2 Outcome

Patients with diagnosed diabetes were defined based on diagnosis by a physician as a part of the program, self-reported diabetes status, and/or self-reported prescription of antidiabetic medication. Undiagnosed diabetes was defined as elevated blood sugar levels without self-reported diabetes status and prescribed antidiabetic medication. In most cases, fasting glucose levels were obtained in which case elevated blood sugar was defined as a blood glucose level of ≥7.00 mmol/l (13). For non-fasting samples, the threshold was set at ≥11.1 mmol/l.

### 2.3 Predictors

The following variables were included in the present analysis: age (years), sex (male/female), blood glucose level (mmol/l), cholesterol level (mmol/l), blood pressure (mmHg), self-reported weight (kg) and height (m), comorbidities (any cardiovascular disease, cancer, kidney disease, gastrointestinal disease, respiratory disease, musculoskeletal diseases, mental disease, and/or other disease), family history of diabetes (yes/no), physical activity (≥150 minutes/week vs. <150 minutes/week), present smoking status (yes/no), and Alcohol Use Disorder Identification Test (AUDIT) score (14). Body mass index (BMI, kg/m^2^) was calculated based on self-reported weight and height. Number of comorbidities were collated into dichotomous variables (none vs. ≥1). Physical activity was defined as any activity resulting in an increase in pulse and respiration rate, such as sports, walking, or bicycling. To assess diet, information was gathered for consumption frequency (never, less than monthly, monthly, weekly, daily once, daily more than once) of the following food items/ingredients: white wheat products, wholewheat products, vegetables, fruits, meats, desserts, and snacks. To increase statistical power, variables on diet were collapsed into dichotomous variables (daily vs. <daily). European Risk Systematic Coronary Risk Evaluation (SCORE) was calculated by taking into consideration the sex, age, smoking status, systolic blood pressure, and cholesterol levels of participants (15). The SCORE could also be calculated for patients with non-fasting blood samples, as cholesterol is not significantly affected by recent nutrition (16). Information on self-reported participation within the last two years in cervical cancer, breast cancer, and colon cancer screening tests were also obtained.

### 2.4 Statistical analysis

Descriptive statistics were presented as absolute numbers and percentages within the whole sample for diagnosed and undiagnosed diabetes. Absolute numbers and percentages within individuals with diagnosed diabetes were presented for type of antidiabetic medication prescribed along with the occurrence of monotherapy, combination therapy, and not receiving any therapy.

For the analysis on health-related variables and lifestyle factors, descriptive statistics were presented as absolute numbers and percentages by diseases status (diagnosed diabetes, undiagnosed diabetes, healthy participants). Patients with diagnosed and undiagnosed diabetes were compared to healthy participants (reference group) by conducting linear regression for continuous variables and multinomial logistic regressions for categorical variables. Unadjusted mean differences (MD) and odds ratios (OR) were computed with 95% Confidence Intervals (95% CIs). Analyses were then adjusted for age and sex resulting in adjusted mean differences (aMD) and adjusted odds ratios (aOR) along with 95% CIs.

The analyses for screening participation were conducted with binary logistic regression models stratified by sex. Patients with diagnosed diabetes were compared to healthy participants (excluding individuals with undiagnosed diabetes from the analysis). Since national age recommendations differ by screening test, we restricted our study population to 40–65 years for the analysis on cervical cancer screening, 45–65 years for breast cancer screening, and 50–70 years for colorectal cancer screening (17). Unadjusted and adjusted ORs for age were computed with 95% CIs.

All analyses were conducted with SPSS 28.0.0. Statistical significance was set at p<0.05.

## 3 Results

A total of 246 individuals took part in the present study. Of the 246 participants, 33 were coded as individuals with diagnosed diabetes, 11 as individuals with undiagnosed diabetes, and 202 as healthy participants, resulting in 17.9% of the population being affected by diabetes overall.

Of the total participants with diabetes (n=44), 25.0% (n=11) were undiagnosed. Out of the participants with diagnosed diabetes (n=33), 75.8% (n=25) were prescribed metformin, 15.2% (n=5) sulfonylurea, 3.03% (n=1) Dipeptidyl Peptidase 4 inhibitors (DPP-4), while 12.1% (n=4) reported to be taking Sodium-glucose Cotransporter-2 inhibitors (SGLT-2). Approximately 12.1% (n=4) of patients with diagnosed diabetes received insulin. Monotherapy was prescribed for 57.6.% (n=19) of patients, while 27.3% (n=9) received medications in combination, and 15.1% (n=5) of patients reported no medication use (see **Table 1**).

**Table 1:** Diagnosis and treatment of patients with diabetes (n=44).

	n (%)
Undiagnosed diabetes	11 (25.0)
Diagnosed diabetes	33 (75.0)
Medication	
Insulin*	4 (12.1)
Non-Insulin*	
Metformin	25 (75.8)
Sulfonylurea	5 (15.2)
DPP-4	1 (3.0)
SGLT-2	4 (12.1)
Treatment regimen*	
No therapy	5 (15.1)
Monotherapy	19 (57.6)
Combination	9 (27.3)

DPP-4, Dipeptidyl Peptidase 4 inhibitor; SGLT-2, Sodium-glucose Cotransporter-2 inhibitor.

*Percentages were calculated within patients with diagnosed diabetes.

Our unadjusted analyses of lifestyle factors and health-related variables revealed that compared to healthy individuals, participants with diagnosed diabetes were significantly older (MD: 4.82, 95% CI: 2.20–7.45), were more likely to have one or more comorbidities (OR: 3.47, 95% CI: 1.37–8.78), consumed whole wheat more often (OR: 2.83, 95% CI: 1.24–6.47), but were less likely to consume desserts daily (OR: 0.27, 95% CI: 0.13–0.60) and were less likely to report good subjective health (OR: 0.27, 95%: 0.12–0.64). After adjustment for age and sex, participants with diagnosed diabetes were still more likely to have one or more comorbidities (aOR: 3.50, 95%: 1.34–9.18), were more likely to be related to individuals with diabetes (aOR: 2.51 95% CI: 1.01–6.24), consumed vegetables more often (aOR: 2.49, 95% CI: 1.07–5.78), desserts less often (aOR: 0.33, 95% CI: 0.15–0.75), and were also less likely to consider themselves in good health (0.28, 955 CI: 0.12–0.67) than healthy participants. Our unadjusted analysis comparing patients with undiagnosed diabetes to healthy participants revealed that patients with undiagnosed diabetes had higher AUDIT scores (MD: 2.88; 95% CI: 0.31 to 5.44). After adjustment, patients with undiagnosed diabetes were more likely to be related to someone with diabetes (aOR: 3.88; 95% CI: 1.01 to 14.9) and still had significantly higher AUDIT scores (aMD 2.75; 95% CI: 0.33 to 5.18) than healthy participants. Both unadjusted and adjusted mean difference of glucose levels were significantly higher for patients with undiagnosed and diagnosed diabetes than healthy participants (see **Table 2**).

**Table:** Table 2 Health-related and lifestyle habits of individuals with diabetes compared to healthy participant.

	Healthy	Undiagnosed	Diagnosed	Undiagnosed vs. Healthy		Diagnosed vs. Healthy	
	(n=202)	(n=11)	(n=33)	OR/MD (95% CI)	aOR/aMD (95% CI)^+^	OR/MD (95% CI)	aOR/aMD (95% CI)^+^
**Age (years)**†	53.2 (7.4)	56.2 (5.7)	58.1 (5.7)	2.94 (-1.39 to 727)	–	4.82 (2.20 to 7.45)***	–
Female	86 (43.6)	4 (36.4)	8 (24.2)	0.77 (0.22 to 2.72)	–	0.43 (0.19 to 1.00)	–
**Glucose (mmol/l)**†	5.63 (1.0)	11.4 (9.9)	7.98 (3.3)	5.77 (4.21 to 7.33)***	5.80 (4.23 to 7.37)***	2.32 (1.35 to 3.30)***	2.38 (1.37 to 3.39)***
Cholesterol (mmol/l)†	5.06 (1.2)	4.60 (0.8)	5.14 (1.5)	-0.46 (-1.50 to 0.58)	-0.46 (-1.51 to 0.59)	0.09 (-0.45 to 0.62)	0.09 (-0.47 to 0.64)
Systolic blood pressure (Hgmm)†	139 (20.8)	138.8 (13.7)	141.6 (22.8)	-0.16 (12.9 to 12.6)	-2.01 (-14.3 to 10.3)	2.57 (-5.16 to 10.3)	-1.24 (-8.94 to 6.47)
European Risk Score (%)†	4.03 (4.3)	5.33 (4.4)	5.52 (4.8)	1.30 (-2.26 to 4.85)	0.44 (-2.48 to 3.35)	1.49 (-0.34 to 3.32)	-0.36 (-1.91 to 1.19)
BMI (kg/m^2^)†	31.0 (20.2)	30.7 (5.1)	31.0 (6.9)	-0.33 (-11.7 to 11.0)	-0.68 (-12.1 to 10.7)	0.02 (-6.87 to 6.91)	-0.76 (-7.88 to 6.36)
**Comorbidity (≥1)**	114 (56.4)	8 (72.7)	27 (81.8)	2.06 (0.53 to 7.99)	1.97 (0.49 to 7.95)	3.47 (1.37 to 8.78)**	3.50 (1.34 to 9.18)*
**Family history of diabetes (yes)**	47 (23.3)	5 (45.5)	10 (30.3)	2.75 (0.80 to 9.41)	3.88 (1.01 to 14.9)*	1.43 (0.64 to 3.23)	2.51 (1.01 to 6.24)*
**Subjective health (good)**	112 (56.0)	3 (27.3)	8 (25.8)	0.30 (0.08 to 1.14)	0.31 (0.08 to 1.23)	0.27 (0.12 to 0.64)**	0.28 (0.12 to 0.67)**
Physical activity (≥150 min)	136 (70.8)	5 (55.6)	18 (62.1)	0.63 (0.12 to 3.23)	0.62 (0.13 to 3.43)	0.90 (0.32 to 2.51)	0.89 (0.31 to 2.59)
Smoking	79 (39.3)	4 (36.4)	15 (45.5)	0.88 (0.25 to 3.11)	0.98 (0.28 to 3.53)	1.29 (0.61 to 2.70)	1.60 (0.74 to 3.49)
**AUDIT score**†	3.12 (3.9)	6.00 (7.2)	3.97 (4.7)	2.88 (0.31 to 5.44)*	2.75 (0.33 to 5.18)*	0.85 (-0.71 to 2.40)	0.40 (-1.11 to 1.91)
White wheat (≥daily)	141 (70.9)	9 (81.8)	19 (59.4)	1.85 (0.39 to 8.83)	1.84 (0.37 to 9.03)	0.60 (0.28 to 1.30)	0.51 (0.23 to 1.17)
**Whole wheat (≥daily)**	30 (15.6)	1 (10.0)	11 (34.4)	0.60 (0.07 to 4.91)	0.51 (0.06 to 4.24)	2.83 (1.24 to 6.47)*	2.25 (0.95 to 5.36)
**Vegetable (≥daily)**	105 (52.2)	7 (70.0)	22 (68.8)	2.13 (0.54 to 8.49)	2.28 (0.56 to 9.22)	2.01 (0.91 to 4.46)	2.49 (1.07 to 5.78)*
Fruit (≥daily)	113 (57.1)	8 (72.7)	20 (62.5)	2.01 (0.52 to 7.79)	1.92 (0.49 to 7.53)	1.25 (0.58 to 2.71)	1.21 (0.54 to 2.68)
Meat (≥daily)	70 (35.7)	4 (44.4)	8 (27.6)	1.44 (0.37 to 5.54)	1.47 (0.38 to 5.68)	0.69 (0.29 to 1.63)	0.74 (0.30 to 1.81)
**Dessert (≥daily)**	132 (65.7)	6 (60.0)	11 (34.4)	0.78 (0.21 to 2.87)	0.85 (0.23 to 3.20)	0.27 (0.13 to 0.60)***	0.33 (0.15 to 0.75)**
Snack (≥daily)	34 (16.9)	2 (20.0)	1 (3.1)	1.23 (0.25 to 6.04)	1.30 (0.26 to 6.53)	0.16 (0.02 to 1.20)	0.16 (0.02 to 1.26)

BMI, Body Mass Index; 95% CI, 95% Confidence Interval; MD: mean difference; aMD, adjusted mean difference; OR, odds ratio; aOR, adjusted odds ratio.

†Continuous variables are reported as mean (SD), mean differences, and 95% Cis.

^+^adjusted for sex and age.

*p<0.05.

**p<0.01.

***p<0.001.

Regarding cancer screening participation rates, no statistically significant differences were observed for either sex between patients with diagnosed diabetes and healthy participants (see **Table 3**).

**Table 3:** Participation in population-level screening tests of patients with diabetes compared to healthy participants.

	Participants with diagnosed diabetes (%)	Healthy participants (%)	OR (95% CI)	aOR (95% CI)*
Females				
Cervix cancer screening (n=94)*	4 (50.0)	43 (51.8)	0.93 (0.22 to 3.97)	1.09 (0.23 to 5.31)
Breast cancer screening (n=91)**	2 (25.0)	40 (46.5)	0.26 (0.07 to 2.01)	0.25 (0.07 to 2.02)
Colon cancer screening (n=56)**	2 (4.17)	0	N/A	N/A
Males				
Colon cancer screening (n=105)**	4 (17.4)	8 (9.8)	1.95 (0.53 to 7.16)	1.85 (0.50 to 6.87)

95% CI, 95% Confidence Interval; N/A, not applicable; OR, odds ratio; aOR, adjusted odds ratio for age.

*OR was adjusted for age.

**Since national age recommendations differ by screening test, we restricted our study population to 40–65 years for the analysis on cervical cancer screening, 45–65 years for breast cancer screening, and 50–70 years for colorectal cancer screening.

## 4 Discussion

In our study, we aimed to investigate the diagnosis rates of diabetes along with the medication, lifestyle habits, and participation in screening tests of patients with diagnosed and undiagnosed diabetes in a highly deprived region of Hungary. Compared to data obtained from the 10th edition of the International Diabetes Federation’s (IDF) Diabetes Atlas, diabetes proportion in the Ormánság region was higher than national data (17.9% vs. 7%) (1, 18). The proportion of undiagnosed diabetes (25%) was also higher than the Hungarian average of 16.7% (1), but compared to global estimates, these results are still within the reported range of values, as prevalence of undiagnosed diabetes varies between 16.7–65.2% in high income countries (19). The relatively higher occurrence of both diabetes overall and undiagnosed diabetes in our study is not surprising, as diabetes occurs more often in deprived areas and recognition of diabetes can also be hindered by low socioeconomic status (20).

Our study revealed two factors that may influence diagnosis rates of diabetes, namely more advanced age and having one or more comorbidities. These two factors may be connected, as several non-communicable diseases are linked to advanced age. Our results suggest that diabetes is rarely diagnosed as a first disease and is more likely to be discovered in patients suffering from other diseases, such as cardiovascular diseases, as a part of their risk assessment. This is indirectly confirmed by the fact that patients with undiagnosed diabetes did not differ from healthy participants regarding comorbidities. It must be noted, however, that the proportion of patients with undiagnosed diabetes affected by one or more comorbidities is similar to patients with diagnosed diabetes, and the lack of significance for patients with undiagnosed diabetes may be linked to their overall lower statistical power. Thus, the reason for having undiagnosed diabetes in this population may reside elsewhere, for instance them being a less health-conscious group of individuals, as indirectly indicated by their higher AUDIT scores.

Since undiagnosed diabetes is associated with worse disease outcomes (21, 22), socially deprived populations should be targeted with diabetes screening programs to increase early recognition of diabetes and initiate therapy and lifestyle modification. Metformin is often considered as the first choice medication for the treatment of most diabetes cases (23). In our study, we found that metformin was prescribed in 75.8% of the cases, which is above the national average of 66% (24), but still somewhat lower than in other European countries, where metformin prescription rates may reach 83% (25). The use of SGLT-2 medication (12.1%) was also above the national average of 8.9% based on data obtained between 2019 and 2022 (26). The prescription rate of SGLT-2 inhibitors is still suboptimal and much lower than in certain European countries, despite the growing evidence on the benefits of using these kinds of drugs (27). Novel antidiabetics, such as SGLT-2 inhibitors, are often used because of their glucose lowering effect with additional positive effects on both blood pressure and bodyweight (28). Moreover, these medications also improve cardiovascular outcomes in patients with established atherosclerosis and decrease their mortality (28). Since comorbidities occurred more often among those with diagnosed diabetes, and cardiovascular diseases are one of the most frequent diseases in Hungary, great population-level benefits could be reaped by increasing the prescription rates of these newer medications (27). In our opinion, the relatively common use of these more modern antidiabetics in this financially deprived population can be related to the universal health insurance of Hungary’s health care system.

Recognizing diabetes is important not only because of the initiation of therapy but also because a diagnosis may encourage some patients to lead a healthier lifestyle, as seen in other studies (29). In our investigation, individuals with diagnosed diabetes consumed vegetables and whole grain significantly more often and desserts significantly less often than healthy participants, while patients with undiagnosed diabetes were not significantly different in this aspect from the healthy population. Moreover, when examining point estimates of patients with undiagnosed diabetes, we found that they consumed white wheat and desserts relatively more often and wholegrain wheat less often than healthy participants. The unhealthy diet of these patients with undiagnosed diabetes may partially explain their higher blood glucose level and predispose them to the progression of their disease. These results imply that by increasing recognition rates, individuals newly diagnosed with diabetes may realize the importance of lifestyle changes, which may help them achieve their goals.

Social deprivation may also negatively impact other health behaviors, such as participation in cancer screening programs (30–32). In our study, participation in cervix screening was similar, around 50%, for those with diagnosed diabetes and healthy participants. Participation rates exceeded the average Hungarian participation rate of 22.5%, as measured between 2008 and 2021 (33), and was on par with the EU mean of 50.7% (34). A possible explanation for the higher participation rate can be that screening tests were performed on-site of community-wide health promotion events, supporting the importance of bringing screening programs closer to the people. Conversely, participation in breast screening was somewhat lower for patients with diabetes (25%) than healthy participants (46.5%). Even though the difference was not statistically significant, possibly due to the lack of statistical power, the results still suggest that patients with diabetes should be targeted and encouraged to take part in breast cancer screening, as cancer occurrence may be higher in patients with diabetes (35). It must be also noted that participation of healthy participants in breast cancer screening was similar to that of the general Hungarian population (48.1–51.5%) (36), but somewhat lower than the EU average of 60.2% (34), further supporting the importance of organizing screening-based events in these communities. Population-level colorectal cancer screening program has not yet been implemented nationally in Hungary even though the pilot program has been launched successfully in certain counties (37). The national participation rate in colorectal cancer screening over the past 10 years was between 5.1–6.8% in Hungary (38). The participation rate of males in our population surpassed the national average but was far lower than the EU mean of 38.2% (34). Conversely female participation rate was comparable (3.3%) to national rates, and neither reached the cost-effective threshold of 46% coverage either (39). Thus, participation in colorectal cancer should be encouraged in Hungary irrespective of disease status.

Limitations of our study includes the use of a convenience sample and lack of representativeness. Based on this, any direct comparison with national statistics should be interpreted cautiously. Another major limitation is the small sample size resulting in underpowered statistics that may have not been able to detect more nuanced differences between study groups. Unfortunately, our study did not examine social deprivation directly either, which may limit the validity of our conclusions. Since both diabetes overall and undiagnosed diabetes tends to peak at later ages (40, 41), this may explain the higher occurrence of diabetes and undiagnosed diabetes in our population, as all our participants were older than 40 years. Another factor confounding our observations regarding the frequency of diabetes diagnosis is healthy volunteer bias. Even though attempts were made to include participants that were harder to reach, participation in our study was still voluntary, and thus our population is likely to consist of healthier and more health-conscious individuals, thus underestimating the true occurrence of undiagnosed diabetes in this region and also affecting our observations made regarding the lifestyle of participants. Moreover, instead of using fasting glucose levels from capillary samples, collecting venous samples and/or also measuring Hba1c levels would have greatly improved the accuracy of our definition of undiagnosed diabetes. Finally, due to the high number of comparisons the occurrence of Type 1 error is inflated.

In our study, we aimed to examine diagnosis rate, therapy, lifestyle, and health behaviors of patients with diabetes in a socially deprived area in Hungary. As expected, we found that the proportion of patients with diabetes overall and undiagnosed diabetes was somewhat higher than the national average. Diagnosis rate was affected by advanced age and having comorbidities, indicating that diabetes may be less often recognized as a first disease. To increase recognition of diabetes, targeted screening tests should be implemented in deprived geographic regions to initiate proper treatment at an early stage of the disease. As a result of the universal health insurance of the Hungarian health care, medication patterns were similar to national tendencies, and the occurrence of novel and somewhat more expensive medications, such as SGLT-2 antagonists, was also comparable to national data in a region consisting of individuals with lower socioeconomic status. Finally, our research indicates that the diagnosis of diabetes is not only important for the timely initiation of therapy, but it can also motivate individuals to lead a healthier lifestyle.

## Data availability statement

The raw data supporting the conclusions of this article will be made available by the authors, without undue reservation.

## Ethics statement

Ethical approval was not required for the studies involving humans because no personal level data was obtained, data was anonymized. The studies were conducted in accordance with the local legislation and institutional requirements. The participants provided their written informed consent to participate in this study.

## Author contributions

KP: Data curation, Formal analysis, Investigation, Writing – original draft, Writing – review & editing. DM: Data curation, Formal analysis, Investigation, Writing – original draft, Writing – review & editing. ND: Writing – original draft, Writing – review & editing. VF: Writing – original draft, Writing – review & editing, Conceptualization, Data curation, Formal analysis, Investigation, Methodology. AT: Conceptualization, Methodology, Writing – review & editing. ZU: Writing – review & editing. IH: Writing – review & editing. IB: Writing – review & editing. ÉP: Writing – review & editing. TB: Writing – review & editing. GF: Writing – review & editing. ZL: Writing – review & editing. MF: Writing – review & editing. ZS: Writing – review & editing.
